# Editorial: Brain-Liver Axis and Glutamate Homeostasis

**DOI:** 10.3389/fnins.2022.879227

**Published:** 2022-04-20

**Authors:** Catya Jiménez-Torres, Mustapha Najimi, Arturo Ortega

**Affiliations:** ^1^Laboratorio de Neurotoxicología, Departamento de Toxicología, Centro de Investigación y de Estudios Avanzados del Instituto Politécnico Nacional (Cinvestav-IPN), Mexico City, Mexico; ^2^Laboratory of Pediatric Hepatology and Cell Therapy, Institut de Recherche Expérimentale et Clinique (IREC), Pôle de recherche hépato-gastroentérologie, Université Catholique de Louvain, Brussels, Belgium

**Keywords:** GLT-1/EAAT2, glutamine synthetase, hyperammonemia, hepatic failure, glial cells, neurocognitive disorder, GLAST/EAAT1

Glutamate has long been recognized as the main excitatory neurotransmitter. It requires the involvement of both neurons and glial cells to elicit its function as a neurotransmitter, in what has been known as a tripartite synapse (Martínez-Lozada and Ortega, 2015). Glutamate in the synaptic cleft is tightly regulated mostly by glial transporters, EAATs, avoiding overstimulation of glutamate receptors and preventing an excitotoxic insult (Danbolt, 2001). Glutamate taken up by astrocytes is converted to glutamine by glutamine synthetase and released and internalized by neurons to be converted back to glutamate through the glutamate/glutamine shuttle (McKenna et al., 2012; Martínez-Lozada and Ortega, 2015). The balance in the components of this machinery is essential for appropriate neurotransmission involving this amino acid. However, the glutamate/glutamine shuttle and the astrocyte-neuron lactate shuttle models support the notion that glutamate is also involved in cell energy metabolism, glutamine synthesis, and ammonium detoxification. Hyperammonemia and acute or chronic hepatic failure may induce alterations in glutamatergic neurotransmission, leading to neuronal damage with motor, cognitive, and behavioral imbalance. The aim of this Research Topic is to provide an update on the relevant role of glutamate, its metabolism, and recycling to understand the molecular mechanisms in the etiology of hepatic failure.

The relevance of glutamate as a key amino acid to cellular signaling in both mammals and invertebrates is approached in the first chapter of this issue. Ortega and Olivares-Bañuelos review the relevance of glial cells in the processes of evolution, development, and regeneration. This approach demonstrated that vertebrate and invertebrate glial cells share both morphological and physiological characteristics, participating in osmoregulation, neuroprotection, neurogenesis, and neurodegeneration processes.

Accurate glutamate-dependent neuronal communication is mediated through glutamatergic receptors which include ionotropic receptors such as NMDARs (N-methyl-D-aspartate receptors) and AMPARs (α- amino-3-hydroxy-5-methyl-4-isoxazole propionic acid receptors). Govindarajulu et al. focused their study on the demonstration that elevated levels of trimethylamine N-oxide, a gut microbe-dependent hepatic metabolite, are associated with impairment of long-term potentiation and synaptic plasticity. The ER stress-mediated PERK signaling pathway is involved in this effect. Their findings provide new evidence to elucidate the possible mechanism involved in mild cognitive impairment and Alzheimer's disease dementia in patients with metabolic disorders.

Acute and chronic liver damage is associated with the development of neurological disorders, promoting different degrees of hepatic encephalopathy. Increased neurotoxin levels impair neurotransmission, change brain energy metabolism, trigger systemic inflammatory response, and alter the blood-brain barrier, key features in the pathophysiology of hepatic encephalopathy. The work of Limón et al. addresses the most recent advancements in our understanding of the dysregulation of glutamatergic neurotransmission in hyperammonemia episodes. The authors point out the glutamate re-uptake as a vital component in synaptic plasticity and function, which fits with a contributory role in cognitive and motor alterations.

All this is complemented by the *ex vivo* study by Voss et al. which demonstrates the prominent role of glutamate dehydrogenase on ammonia fixation during hyperammonemia. Since a compensatory increase in alanine production catalyzed by the concerted action of glutamate dehydrogenase and alanine aminotransferase has been demonstrated when glutamine synthetase is inhibited (Dadsetan et al., 2013). Voss et al. point out that glutamate dehydrogenase upholds *de novo* glutamate synthesis from glucose and by these means provides a substrate for glutamine production. Glutamate dehydrogenase is coupled to alanine aminotransferase which operates in a cyclic manner to fixate ammonia and subsequently regenerate α-ketoglutarate from glutamate, and simultaneously produces alanine which is capable of leaving the brain. This constitutes a novel insight into the pathway for ammonia elimination from the brain in the form of alanine.

To understand the role of glutamate through the brain-liver axis as a direct/indirect target to promote the imbalance in the central nervous system, Jiménez-Torres et al. demonstrate that the disruption of glutamate transporters in the liver-brain axis occurs at the early stages of acute liver injury. Significantly, acute liver damage is associated with neuronal cell loss, glial activation, and imbalance in the expression of key proteins in glutamate recycling, such as glutamate transporters and glutamine synthetase mainly in the cerebellum, suggesting a short-term regulation of glutamate transporters in the central nervous system. Neuronal cell loss reported in this study supports the results from an interesting meta-analysis by López-Franco et al. which indicates that cirrhotic patients with a history of hepatic encephalopathy show clear cognitive deficits compared to control cirrhotic patients and that these differences are not fully restored after liver transplant.

In conclusion, the articles presented in this e-book collect important contributions from different experts in the field of glutamate homeostasis that begin to characterize brain-liver crosstalk in hepatic failure.

## Author Contributions

All authors listed have made a substantial, direct, and intellectual contribution to the work and approved it for publication.

## Funding

AO would like to acknowledge funding from the CONACYT (No. 255087).

## Conflict of Interest

The authors declare that the research was conducted in the absence of any commercial or financial relationships that could be construed as a potential conflict of interest.

## Publisher's Note

All claims expressed in this article are solely those of the authors and do not necessarily represent those of their affiliated organizations, or those of the publisher, the editors and the reviewers. Any product that may be evaluated in this article, or claim that may be made by its manufacturer, is not guaranteed or endorsed by the publisher.
